# An Immunohistochemistry Study of Sox9, Runx2, and Osterix Expression in the Mandibular Cartilages of Newborn Mouse

**DOI:** 10.1155/2013/265380

**Published:** 2013-05-16

**Authors:** Hong Zhang, Xiaopeng Zhao, Zhiguang Zhang, Weiwei Chen, Xinli Zhang

**Affiliations:** ^1^Department of Orthodontics, Guanghua School of Stomatology, Sun Yat-Sen University, Guangzhou 510055, China; ^2^Dental and Craniofacial Research Institute, School of Dentistry, University of California, Los Angeles, CA, 90095, USA; ^3^Department of Oral and Maxillofacial Surgery, Sun Yat-sen Memorial Hospital, Sun Yat-Sen University, Guangzhou 510120, China; ^4^Department of Oral and Maxillofacial Surgery, Guanghua School of Stomatology, Sun Yat-Sen University, Guangzhou 510055, China; ^5^Institute for Medical Biology, College of Life Sciences, South-Central University for Nationalities, Wuhan 430074, China

## Abstract

The purpose of this study is to investigate the spacial expression pattern and functional significance of three key transcription factors related to bone and cartilage formation, namely, Sox9, Runx2, and Osterix in cartilages during the late development of mouse mandible. Immunohistochemical examinations of Sox9, Runx2, and Osterix were conducted in the mandibular cartilages of the 15 neonatal C57BL/6N mice. In secondary cartilages, both Sox9 and Runx2 were weakly expressed in the polymorphic cell zone, strongly expressed in the flattened cell zone and throughout the entire hypertrophic cell zone. Similarly, both transcriptional factors were weakly expressed in the uncalcified Meckel's cartilage while strongly expressed in the rostral cartilage. Meanwhile, Osterix was at an extremely low level in cells of the flattened cell zone and the upper hypertrophic cell zone in secondary cartilages. Surprisingly, Osterix was intensely expressed in hypertrophic chondrocytes in the center of the uncalcified Meckel's cartilage while moderately expressed in part of hypertrophic chondrocytes in the rostral process. Consequently, it is suggested that Sox9 is a main and unique positive regulator in the hypertrophic differentiation process of mandibular secondary cartilages, in addition to Runx2. Furthermore, Osterix is likely responsible for phenotypic conversion of Meckel's chondrocytes during its degeneration.

## 1. Introduction

The development of cartilages plays a pivotal role in the development and growth of the mandible. Mandibular cartilages are derived from ectomesenchymal cells of the first pharyngeal arch, but their characteristics differ. Meckel's cartilage is a fetal cartilaginous skeleton in the mandible. Although it is classified as primary cartilage similar to limb bud cartilage, it contains four distinct portions, each having a different fate. The anterior, intermediate, and proximal portions convert to intramandibular symphysis, sphenomandibular ligament, and the inner ear ossicles, respectively. The posterior portion of intramandibular Meckel's cartilage facing the developing molar buds undergoes developmental events similar to endochondral ossification, but the degradation mechanisms of this portion are distinct from those in endochondral ossification [1]. Independent of the chondroskeleton, four secondary cartilages including the condylar, coronoid, angular, and symphyseal cartilage strongly influence the further development of the mandible. These secondary cartilages differ from the primary cartilage in embryonic origin, morphological and biochemical organization. They are derived from the periosteum of intramembranous bone after (secondary to) bone formation [2, 3]. Furthermore, they display a unique mode of cell proliferation and differentiation. The condylar cartilage, as a principle secondary cartilage, does not form columns of proliferating chondrocytes and grows multidirectionally to adapt to the mandibular fossa of the temporal bone [4]. 

 Recent studies showed that the three master transcription factors of Sox9, Runx2, and Osterix are involved in the formation of Meckel's cartilage and mandibular condylar cartilage [3, 10]. Sox9, Runx2, and Osterix are key transcription factors, which are necessary in skeletal cell fate decision [11]. Sox9 (SRY-box containing gene 9) is an essential and nonredundant factor of chondrogenesis. Analyses in genetically modified mice revealed that Sox9 promotes the early stage, but suppresses the terminal stage of chondrocyte differentiation in limb bud cartilage [12–14]. On the contrary, the multifunctional transcription factor Runx2, which is expressed in prehypertrophic and hypertrophic chondrocytes, is a main positive regulator of hypertrophic differentiation in late chondrogenesis of the limb buds [5, 15, 16]. New in vitro data demonstrated that Sox9 negatively regulates Runx2 by enhancing Bapx1 expression, which leads to the inhibition of terminal chondrocyte differentiation [17]. Osterix, which acts downstream of Runx2 during bone formation, is expressed in chondrocyte progenitors and prehypertrophic chondrocytes in rib, spine, and limb cartilages, suggesting that Osterix may play a critical role during the primary cartilage maturation in combination with Runx2 and Sox9 [6, 7].

 However, the transcriptional control of the later development of mandibular cartilages remains poorly understood. At birth, the rostral process of intramandibular Meckel's cartilage is undergoing endochondral ossification, while the posterior portion of intramandibular Meckel's cartilage is degenerating [18–20]. Meanwhile, four secondary cartilages, especially the condylar cartilage, were not well documented in terms of their developmental characteristics, although they function mainly as a growth cartilage similar to limb bud cartilage. At present, transcription factors are attracting increasing clinical attention because of their roles in the etiology and pathogenesis of malformations and growth disorders, degenerative diseases, and in regenerative and repair processes [21, 22]. The findings that Runx2-deficient mice lack mandibular condylar cartilage and had deformed Meckel's cartilage indicate that Runx2 is essential for the formation of the mandibular cartilages [23]. In many cleidocranial dysplasia (CCD) patients who were link to Runx2 deficent, however, there are no abnormal findings in the mandible, in spite of cases of condylar malformation, persistent symphysis, or a narrow coronoid process being also known [24, 25]. These investigations provided a hint that Runx2 may be just one of essential biological factors influencing the development and growth of mandibular cartilages. The present study is to examine tissue distribution of Sox9, Runx2, and Osterix in newborn mice mandibular cartilages using immunohistochemistry technique and investigate whether these transcription factors have similar functions to those in limb bud cartilage which will contribute to current understanding of mechanisms of the development of mandible and the possible pathogenesis of some craniofacial anomalies involving mandible.

## 2. Materials and Methods

All animals were housed and handled in accordance with guidelines of the Chancellor's Animal Research Committee of the Office for Protection of Research Subjects at the University of California, Los Angeles, CA, USA.

### 2.1. Tissue Preparation

A total of 15 newborn C57BL/6N mice were collected in 2 hours right after being delivered and used for this study. The mandibles were then removed and immersed in 4% paraformaldehyde (0.1 M phosphate buffer, pH 7.4) for 1 day at 4°C. The specimens were decalcified with 10% EDTA for 5 days at 4°C and then embedded in paraffin using standard procedures. Sections (5 *μ*m) were cut in the plane parallel to the ascending ramus of the mandible, all the mandibular cartilages being in one section. For general morphology, deparaffinized sections were stained with hematoxylin and eosin. The skeletal staining with Alizarin Red-Alcian Blue was performed for preparation of gross specimen of mandible as reported previously [26]. 

### 2.2. Immunohistochemistry

Five-micron-thick paraffin sections were dewaxed in xylenes and rehydrated in ethanol baths. Endogenous peroxidases were blocked by incubating sections in 3% hydrogen peroxide for 20 min at room temperature. Sections were incubated with anti-Runx2, anti-Osterix, and anti-Sox9 primary antibodies (Santa Cruz Biotechnology, CA, USA) (dilution 1 : 100) and biotinylated anti-rabbit or anti-goat IgG secondary antibody (Vector Laboratories, Burlingame, CA) for 1 h at room temperature. Positive immunoreactivity was detected using Vectastain ABC kit (Vector Laboratories, Burlingame, CA, USA) and AEC chromogenic substrate (Dako, Carpiteria, CA, USA) with red positive staining. A negative control was performed by replacing primary antibody solutions with PBS. Sections were counterstained with hematoxylin for 30 sec followed by rinsing 5 min in running water. Photomicrographs were acquired using an Olympus BX51. Image-pro Plus 6.0 software was used to calculate stained area and Integrated Optical Density (IOD). The average optical density (mean density) represented the intensity of protein expression and was counted in 4 random fields (×20 objective) of each cartilage area and trabecular bone area per section. The mean density is equal to (IOD SUM)/area. For exact analysis, three sections were prepared at similar plane for each sample. ANOVA was used for multiple groups' comparison, and Student's *t*-test was used for comparison between any two groups. Statistical significance defined as *P* < 0.05. 

## 3. Results

### 3.1. Histological Analysis of Cartilages in Newborn Mouse Mandible

**Figure 1:**
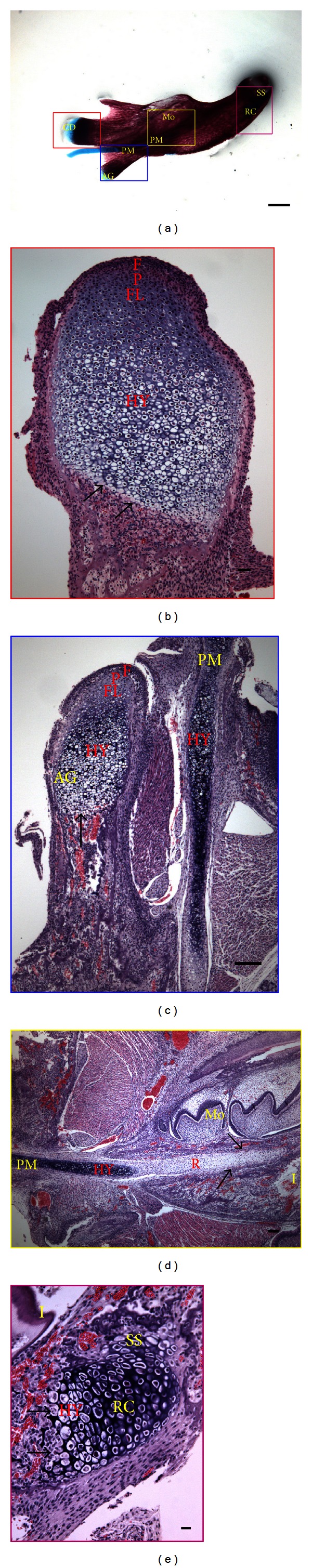
Histological analysis of mandibular cartilages of mice at newborn stage. (a) Lingual view of mandible by Alizarin Red and Alcian Blue staining; (b) hematoxylin-eosin sections of condylar cartilage, and (c) hematoxylin-eosin sections of angular cartilage (AG) similarly displaying four different cell zones: a thin fibrous cell zone (F), a polymorphic cell zone (P), a wider flattened cell zone (FL), and a broad hypertrophic cell zone (HY); (d) hematoxylin-eosin staining pattern of the matrix of the posterior portion of intramandibular Meckel's cartilage (PM) changed from the intense hematoxylin to the light eosin in the resorption (R) facing the molar buds (Mo) and incisor (I); (e) hematoxylin-eosin sections of the endochondral ossification rostral process of Meckel's cartilage (RC) and symphyseal secondary cartilage (SS) facing the incisor (I). Scale bar: 100 *μ*m for (a) and (b), 250 *μ*m for (c) and (d), and 50 *μ*m for (e).

Mandibular cartilages in newborn mouse included the portions of Meckel's cartilage, condylar, angular, and symphyseal secondary cartilage, while cartilage was not present in the coronoid process of the newborn mouse (Figure 1(a)). On the basis of the cellular morphological changes, the mandibular condylar and angular cartilages were histologically composed of four different cell zones: a thin fibrous cell zone, a polymorphic cell zone, a wider flattened cell zone, and a broad hypertrophic cell zone occupied the lower half of the organ (Figures 1(b) and 1(c)). Almost all of the intramandibular bar of Meckel's cartilage had ossified completely, but a small amount of Meckel's cartilage remained in a limited portion of the rostral region and at the mylohyoid groove between the condylar and angular processes. At the posterior portion of intramandibular Meckel's cartilage, HE staining pattern of the matrix changed from the intense hematoxylin to the light eosin in resorption area, which indicated the degradation of Meckel's cartilage matrix during development (Figure 1(d)). Furthermore, the endochondral ossification rostral process of Meckel's cartilage and symphyseal secondary cartilage serve as a chondrogenic mandibular symphysis of newborn mice (Figure 1(e)). The opened chondrocytic lacunae and disconnected cartilaginous matrix (arrows in Figures 1(b), 1(c), and 1(e)) were clearly found in the resorption area of condylar, angular, and symphyseal secondary cartilage, in addition to rostral cartilage, while the appearing perichondrium and the eosinophilic cartilage erode on the lateral sides (arrows in Figure 1(d)) were observed in the posterior portion of intramandibular Meckel's cartilage. These results indicated that the degradation of cartilaginous matrix in the posterior portion of intramandibular Meckel's cartilage is distinct from others among mandibular cartilages. 

### 3.2. Immunohistochemical Analysis of Sox9, Rux2, and Osterix in Cartilages of the Newborn Mouse Mandible

**Figure 2:**
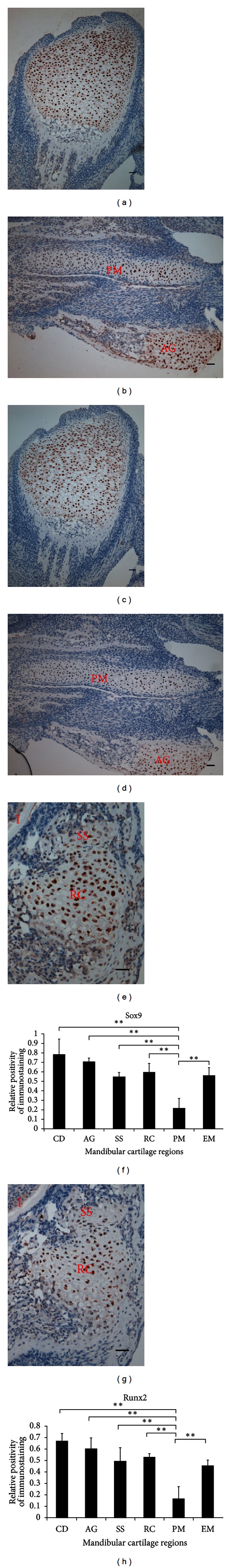
Immunohistochemistry of Sox9 and Runx2 in mandibular cartilages of mice at newborn stage. Sox9 (a, b, and e) and Runx2 (c, d, and g) showed similar expression patterns throughout all the mandibular cartilages. In condylar cartilage (a and c), angular cartilage (b and d), and symphyseal secondary cartilage and rostral cartilage (e and g), both Sox9 and Runx2 were strongly expressed by cells entire hypertrophic cell zone. Scale bar: 100 *μ*m for (a, b, c, and d) and 50 *μ*m for (e and g). Results of Sox9 (f) and Runx2 (h) immunohistochemistry in hypertrophic zones of mandibular cartilages including condylar (CD), angular (AG), and symphyseal secondary cartilage (SS), and rostral process (RC), posterior Meckel's (PM), and extramandibular Meckel's (EM) cartilage were quantitated by average optical density of positive staining per 200 field (**P < 0.001). The expression levels of both transcriptional factors in the posterior portion of uncalcified intramandibular Meckel's cartilage (b and d) were significantly reduced than in other cartilages.

Interestingly, transcription factors Sox9 and Runx2 showed similar expression level and tissue distribution patterns throughout all the mandibular cartilages of newborn mice. In secondary cartilages, both Sox9 and Runx2 were weakly expressed by cells in the polymorphic cell zone, strongly in the flattened cell zone and throughout the entire hypertrophic cell zone. To quantitatively measure changes in expression of transcriptional factors critical for chondrogenic differentiation, Sox9 (Figure 2(f)) and Runx2 (Figure 2(h)) immunohistochemistry in hypertrophic zones of mandibular cartilages at newborn stage were quantitated by average optical density of positive staining. The expression levels of both transcriptional factors in the degrading posterior portion of intramandibular Meckel's cartilage (Figures 2(b) and 2(d)) exhibited a significantly decrease, compared with others among mandibular cartilages. Meanwhile, cells in the rostral cartilage (Figures 2(e) and 2(g)) and cells in extramandibular Meckel's cartilage (Figures 2(f) and 2(h)) similarly expressed both transcriptional factors more than in the degrading posterior portion of intramandibular Meckel's cartilage. Unexpectedly, Sox9, as Runx2, was expressed in all the terminal chondrocytes of the mandibular cartilages in newborn mice (Figures 2(a), 2(b), and 2(e)), contrary to the express pattern of Sox9 in limb bud cartilage [8]. This spatial distribution pattern indicated Sox9's requirement in the terminal stage of mandibular chondrocyte differentiation.

**Figure 3:**
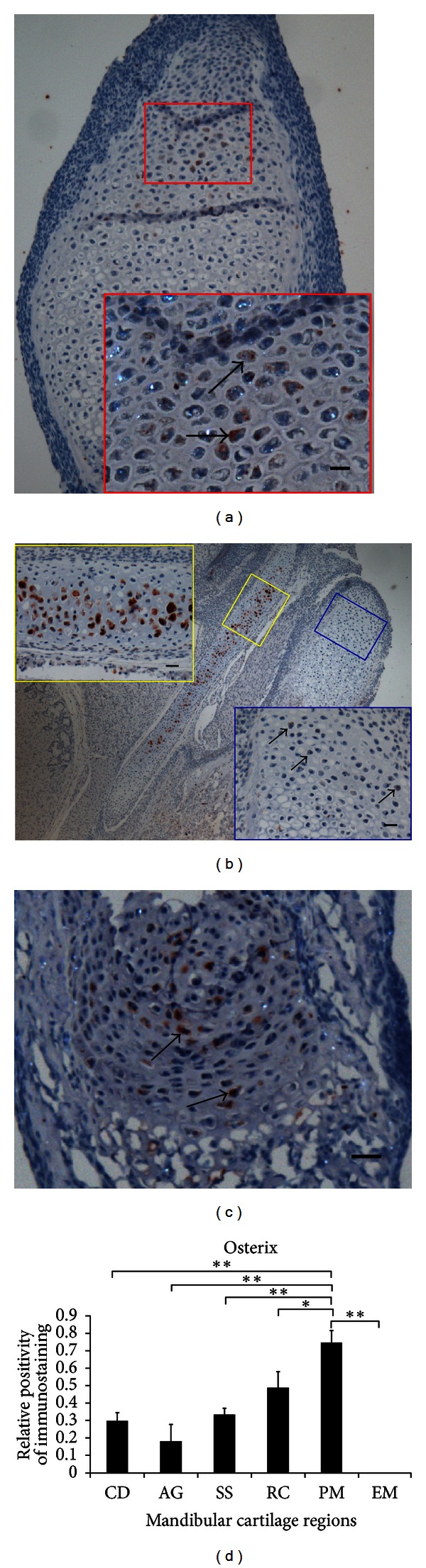
Immunohistochemistry of Osterix in mandibular cartilages of mice at newborn stage. Osterix was at a extremely low level in condylar cartilage (in the red box of (a)) and angular cartilage (in the blue box of (b)) while intensely expressed in the center of the Meckel's cartilage containing the strong basophilic matrix (in the yellow box of (b) and Figure 1(c)). Further, Osterix was moderately expressed in the rostral process (c). Scale bar: 25 *μ*m for (a′, b′, and b′′) and 50 *μ*m for (c). Results of Osterix (d) immunohistochemistry in mandibular cartilages including condylar (CD), angular (AG), and symphyseal secondary cartilage (SS) and rostral process (RC), posterior Meckel's (PM), and extramandibular Meckel's (EM) cartilage were quantitated by average optical density of positive staining per 200 field (*P < 0.05, **P < 0.001). Immunohistochemistry of Osterix in the posterior portion of intramandibular Meckel's cartilage was significantly stronger and had more positive cells than in other cartilages.

 Runx2 and Osterix are involved in the formation of Meckel's cartilage and mandibular condylar cartilage [3, 10]. Thus, we correlated the expression patterns of Runx2 (Figures 2(c), 2(d), and 2(g)) and Osterix (Figure 3) in mandibular cartilages at newborn stages. Results showed that Osterix was at an extremely low level in part of cells of the flattened cell zone and the upper hypertrophic cell zone in condylar cartilage and angular cartilage, independent on Runx2. Unlike Sox9, the spatial pattern of Osterix in condylar cartilage and angular cartilage was consistent with that in limb bud cartilage [7, 9]. Notably, Osterix was intensely expressed only in hypertrophic chondrocytes of the center of the uncalcified Meckel's cartilage containing the strong basophilic matrix, while it was entirely absent in hypertrophic chondrocytes in the resorption area containing the light eosinophilic matrix (Figures 3(b), 1(c), and 1(d)). Additionally, the expression level of Osterix in the hypertrophic chondrocytes of Meckel's cartilage (Figure 3(b)) was significantly higher compared with that in condylar cartilage and angular cartilage which have only few positive cells in the flattened cell zone and the upper hypertrophic cell zone (Figures 3(a), 3(b), and 3(d)). Moreover, Osterix was moderately expressed in part of hypertrophic chondrocytes in the rostral process (Figure 3(c)), while it was absent in extramandibular Meckel's cartilage. At present, the mechanisms of Osterix regulation of chondrocyte differentiation and function are still under investigation, whereas the significantly intense immunohistochemistry of Osterix in hypertrophic chondrocytes of the center of the uncalcified Meckel's cartilage provided evidence of Osterix's role in the degradation of the posterior portion of uncalcified intramandibular Meckel's cartilage.

### 3.3. Comparison of Expression Intensity of Runx2 and Osterix in the Chondrocytes with That in the Osteoblasts of the Newborn Mouse Mandible

**Figure 4:**
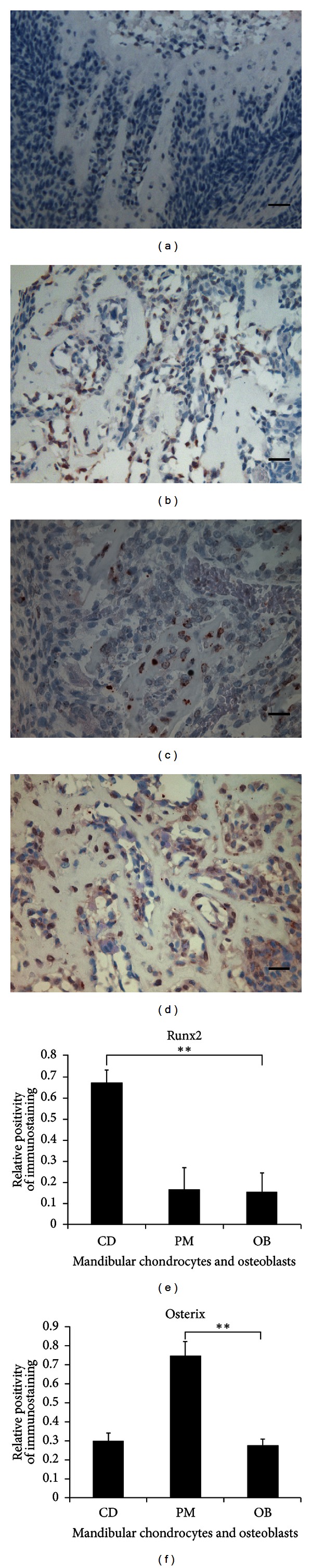
Immunohistochemistry of Runx2 and Osterix in mandibular osteoblasts of mice at newborn stage. Runx2 protein which was expressed in secondary hypertrophic chondrocytes was not localized in the cells gathering in the erosive front of mandibular secondary cartilages (a), but in some osteoblasts surrounding the trabecular bone and some osteocytes buried in the trabecular bone in the mandible (b). The expression of Runx2 protein in condylar hypertrophic chondrocytes was significantly stronger than that in osteoblasts (e). Meanwhile, Osterix was localized in some osteoblasts and bone marrow cells in subchondral bone area (c), while more positive osteoblasts and osteocytes were visualized in the trabecular bone area (d). The immunohistochemistry of Osterix in hypertrophic chondrocytes of the center of the uncalcified Meckel's cartilage is still significantly more intense than that in the osteoblasts (f). (**P < 0.001) CD: condylar cartilage; PM: the posterior portion of intramandibular Meckel's cartilage; and OB: osteoblasts. Scale bar: 25 *μ*m for (a, b, c, and d).

Since Runx2 and Osterix are indispensable for osteoblast differentiation and known to be expressed in osteoblasts, we first confirmed the positive staining of osteoblasts using the same sections of cartilages with Runx2 and Osterix positive staining, which also validated our IHC approach to be highly reliable. Then, we compared the expression intensity of the two key transcriptional factors related to bone formation in chondrocytes with that in mandibular osteoblasts to further confirm the significance of both during the development of mandibular cartilages. Similar to the condylar subchondral bone in 56-day-old rats [27], Runx2 protein which was expressed in secondary hypertrophic chondrocytes was not localized in the cells gathering in the erosive front of all the mandibular secondary cartilages (Figure 4(a)), but in some osteoblasts surrounding the trabecular bone and some osteocytes buried in the trabecular bone in the mandible (Figure 4(b)). Thus, we quantitatively analyzed the expression of Runx2 protein in osteoblasts and osteocytes (Figure 4(e)), comparing with that in condylar cartilage and the posterior portion of intramandibular Meckel's cartilage. In the present study, the expression of Runx2 protein in condylar hypertrophic chondrocytes was the most intense (Figures 2(h) and 4(e)), being statistically significant difference from that in osteoblasts, which indicated an important role of Runx2 in secondary chondrocyte maturation, in addition to that in chondrocyte maturation of growth cartilage and osteoblast differentiation. Expectedly, Osterix was localized in some osteoblasts and bone marrow cells in sub-chondral bone area (Figure 4(c)). Furthermore, more positive osteoblasts and osteocytes were visualized in the trabecular bone area (Figure 4(d)). Interestingly, the immunohistochemistry of Osterix in hypertrophic chondrocytes of the center of the uncalcified Meckel's cartilage is still significantly more intense than that in the osteoblasts (Figure 4(f)). This pointed out that Osterix highly likely performed a regulatory effect on the degradation of the posterior portion of uncalcified intramandibular Meckel's cartilage. 

## 4. Discussion 

The majority of in vivo studies on cartilage differentiation are carried out using the appendicular skeleton as a model system, with the implicit assumption that chondrogenesis is equivalent throughout the body. However, Eames directly tested that the programs of chick head chondrogenesis are unique by comparing the neural crest-derived pharyngeal arch skeleton to that of the mesoderm-derived limb, due to the fact that each skeleton forms from unique embryonic populations [28]. Meckel's cartilage and mandibular secondary cartilages are markedly distinguished from limb bud cartilage in their embryonic origin. The mechanisms that regulate the diverse developmental programs in Meckel's cartilage and mandibular secondary cartilages remain to be discovered. The present study investigated the expression of the essential transcription factors related to chondrogenesis in these cartilages during the later development of mandibular cartilages. 

**Figure 5:**
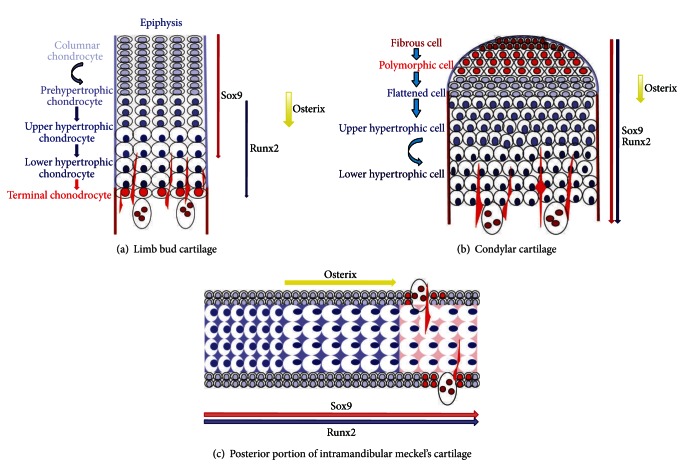
Schematic representations of the expression pattern of three key transcription factors in the different types of cartilage during the newborn stage. The expression pattern of Sox9, Runx2, and Osterix in limb bud cartilage is based on previous reports of Kim et al. [5], Kaback et al. [6], Nishimura et al. [7], Ng et al. [8], and Dy et al. [9]. Furthermore, the expression patterns of Sox9 (red), Runx2 (blue), and Osterix (yellow) in condylar cartilage and Meckel's cartilage are based on the present findings. Long arrows indicate the expressing cell zones of transcription factors in cartilage. (a) limb bud cartilage, (b) condylar cartilage, and (c) the posterior portion of intramandibular Meckel's cartilage.

 The accumulated studies confirmed that Sox9 accelerates chondrocyte differentiation in proliferating chondrocytes but inhibits the terminal stages of chondrocyte differentiation in limb bud cartilage [29, 30]. However, few investigations focused on the mechanism of Sox9 in secondary chondrocyte differentiation [31]. Our findings clearly demonstrated that the key transcription factor Sox9 was strongly expressed at the whole hypotrophic cell zone of condylar cartilage and angular cartilage in newborn mice, which was different from the expression pattern of Sox9 in limb bud cartilage (Figure 5(a)) [8]. Moreover, Rabie et al. have demonstrated that Sox9 were expressed at the hypertrophic cell zone of the condylar cartilage in 36-day-old rats and continued to be expressed throughout the examined period until day 52 [32]. Conversely, in limb bud cartilage, both of the Sox9 transcripts and protein were absent or at very diminished levels in hypertrophic chondrocytes [8, 9]. The recent data demonstrated that Sox9 is a major negative regulator of cartilage vascularization, bone marrow formation, and endochondral ossification [33]. Despite this observation, our investigations indicate that Sox9 downregulation is not necessary in the terminal stage of secondary cartilage development. We speculated that the transcription factor Sox9 may be a main positive regulator in the secondary cartilage terminal maturation, contrary to its function in later differentiation of limb bud cartilage, based on strong expression of Sox9 in the mandibular secondary hypertrophic cell zone. 

 In the present study, surprisingly, transcription factors Sox9 and Runx2 were similarly expressed at mandibular secondary cartilages in newborn mice, suggesting that Sox9 and Runx2 may coregulate secondary chondrocyte differentiation. In avian secondary cartilage formation, Buxton reported that Runx2-expressing preosteoblasts exit from the cell cycle and rapidly differentiate into hypertrophic chondrocytes, which is correlated with the up-regulation of Sox9 [31]. In addition, Buxton described two routes to chondrocyte hypertrophy and had postulated that precursors expressing Sox9 differentiate into prehypertrophic/hypertrophic chondrocytes mediated by the up-regulation of Runx2 in primary cartilage formation. Whereas, preosteoblasts expressing Runx2 differentiate into prehypertrophic/hypertrophic chondrocytes mediated by the upregulation of Sox9 in secondary cartilage formation [31]. Mammalian mandibular secondary cartilages are a heterogeneous tissue containing cells at various stages of chondrocyte maturation [34]. Moreover, these secondary cartilages manifest a unique zone-like packing of maturing chondrocytes [35]. Shibata and Yokohama-Tamaki recently demonstrated that the mandibular secondary cartilage anlages are derived from Runx2 mRNA expressing mandibular anlage [10]. Thus, our observations on the overlapping expression of Sox9 and Runx2 at mandibular secondary cartilages in newborn mice support Buxton's proposed concept in principle. The up-regulation of Sox9 from the polymorphic cell zone to the hypertrophic cell zone might act as a trigger for subsequent mammalian secondary chondrocyte differentiation. This can be interpreted as evidence of a unique differentiation pathway: the formation of secondary hypertrophic chondrocytes from osteoblast precursors, with the help of the positive regulator Sox9.

 More unexpectedly, our finding that Sox9 and Runx2 were coexpressed in the hypertrophic cell zone of the rostral region is not in line with the analyses of endochondral ossification in limb bud cartilage. The previous studies revealed that Sox9 inhibits the hypertrophic chondrocyte differentiation through suppression of Runx2 in endochondral ossification of limb bud cartilage [17]. Furthermore, Sox9 protein needs to be degraded to allow chondrocyte terminal maturation in limb bud cartilage [13]. However, Eames et al. had proposed that a unique combination of Sox9 and Runx2 may drive the expression of the major marker of hypertrophic chondrocytes, Col10, based on the analysis of Sox9 and Runx2 functions in primary cartilage differentiation of the avian cranial skeleton [36]. In the present study, the overlapping expression pattern of Sox9 and Runx2 in the hypertrophic cell zone of the rostral region of Meckel's cartilage provides clear evidence that Runx2 can drive the chondrocyte terminal differentiation in the presence of Sox9 protein. Additionally, our data that Sox9 and Runx2 were similarly expressed less in the hypertrophic cell zone of the posterior portion of intramandibular Meckel's cartilage has reinforced the notion that degeneration of Meckel's cartilage represents a different process from endochondral ossification. 

Normally, Osterix is present at an extremely low level in prehypertrophic chondrocytes of limb bud cartilage, compared to osteoblasts [9]. To our knowledge, the present study is the first to demonstrate the expression of Osterix protein in mandibular cartilages. Osterix protein is faintly expressed in prehypertrophic chondrocytes of secondary cartilages, similar to limb bud cartilage, which suggests that Osterix plays similar roles during the two types of cartilage development. By contrast, Osterix protein is intensely expressed in hypertrophic chondrocytes in the central zone of the bars of intramandibular Meckel's cartilage, while Osterix protein is not present in cells around light eosinophilic matrix dynamically changed from the strong basophilic matrix in the front of the degrading Meckel's cartilage. The light eosinophilic matrix in front of the degrading Meckel's cartilage might display the calcified cartilage matrix [37]. A great amount of in vitro data demonstrated that the chondrocytes of Meckel's cartilage can transdifferentiate to osteogenic cells as characterized by production of type I collagen [38–40]. Furthermore, the previous in vivo investigations revealed that the extracellular matrix of intramandibular portion of the Meckel's cartilage is replaced gradually by type I collagen secreted by chondrocytes during the development of Meckel's cartilage [41]. We speculated that Osterix may be relevant to phenotypic conversion of Meckel's chondrocytes. The enhanced expression of Osterix in mature chondrocytes might be an explanation of type I collagen synthesis by chondrocytes in Meckel's cartilage. Further studies are needed to elucidate the exact role of Osterix during the late development of Meckel's cartilage. On the other hand, the disparity in the expression pattern between Osterix and Runx2 in chondrocytes in the present study, suggested that Osterix might perform its regulation and function in mandibular cartilage development, independent of Runx2. Moreover, with respect to the more remarkable expression of Runx2 in the condylar cartilage and Osterix in intramandibular degrading Meckel's cartilage relative to those in osteoblasts in the present study we speculated that Runx2 or Osterix could need much more intense expression in the chondrocytes than in the osteoblasts, in order to play a functional role during the development of mandibular cartilages. 

 Cartilage is a complex and developmentally important tissue type. Transcriptional factors are crucial to the development of cartilages. The differential expression of key transcriptional factors in several types of cartilages will dictate the distinct cellular events during the development of the cartilages. The present data provide insights into the similar roles that master transcriptional factors Sox9 and Runx2 play during the later development of mandibular cartilages, which is different from that in limb bud cartilage. It is necessary to investigate in further detail whether the differences in cellular events between ectomesenchymal chondrocytes and mesodermal chondrocytes involve the derivation of the cells. Furthermore, Osterix is likely responsible for phenotypic conversion of Meckel's chondrocytes during its degeneration, based on its intensive expression in hypertrophic chondrocytes of the degrading Meckel's cartilage of newborn mice. Human mandibular anomaly appears to be a common malformation and appears in multiple congenital birth defect syndromes, ranging from agnathia (agenesis of the jaw) to micrognathia to patterning malformations. These malformations are particularly devastating, as our faces are our identity [42]. The regeneration of complex facial structures requires precision and specificity. A much more thorough understanding of the mechanism of master transcriptional factors in mandibular chondrogenesis lay the important foundation for the application of targeted interventions at the molecular level, endogenous tissue engineering, and cell-based therapies in mandibular anomalies.

## 5. Conclusions

Our study demonstrated similar tissue distribution of Sox9 and Runx2 in newborn mice mandibular cartilages, which is distinguished from that in limb bud cartilage. It is speculated that Sox9 is a main and unique positive regulator in the hypertrophic differentiation process of mandibular secondary cartilages, in addition to Runx2. Moreover, the distinct expression pattern of osterix in degenerating posterior portion of Meckel's cartilage suggests that Osterix may be relevant to phenotypic conversion of Meckel's chondrocytes.
